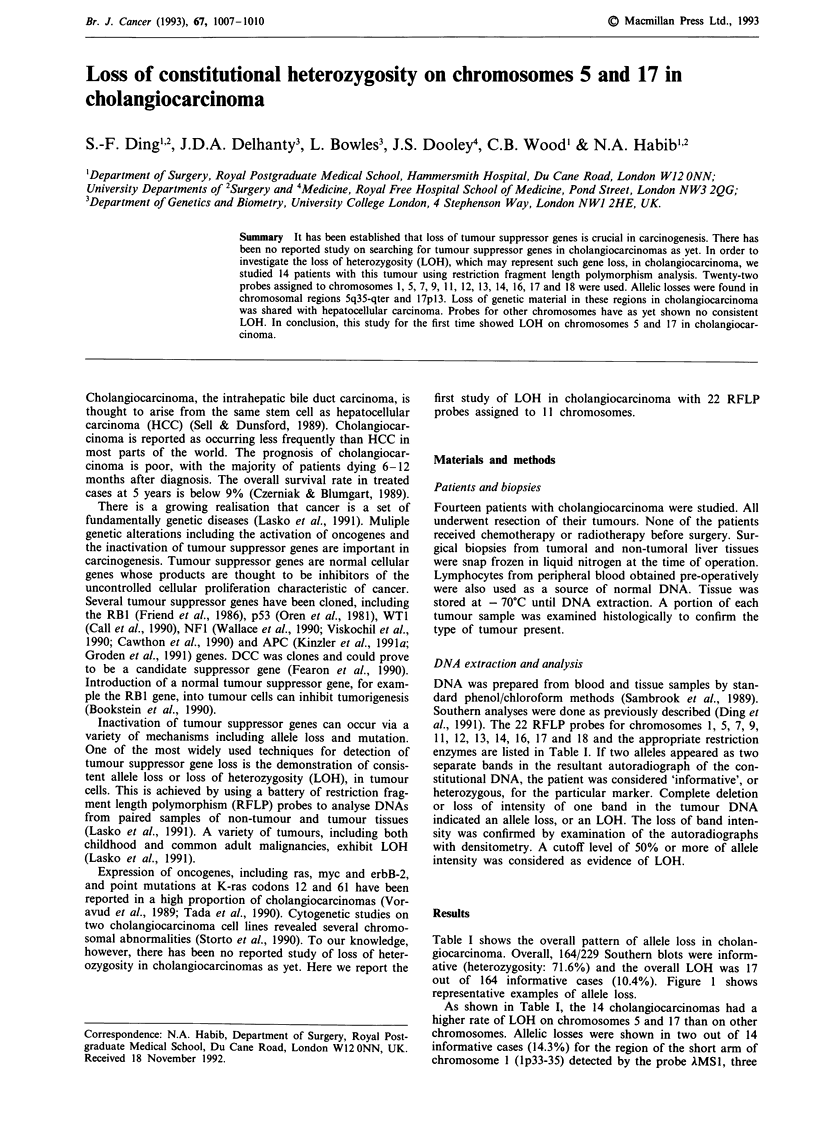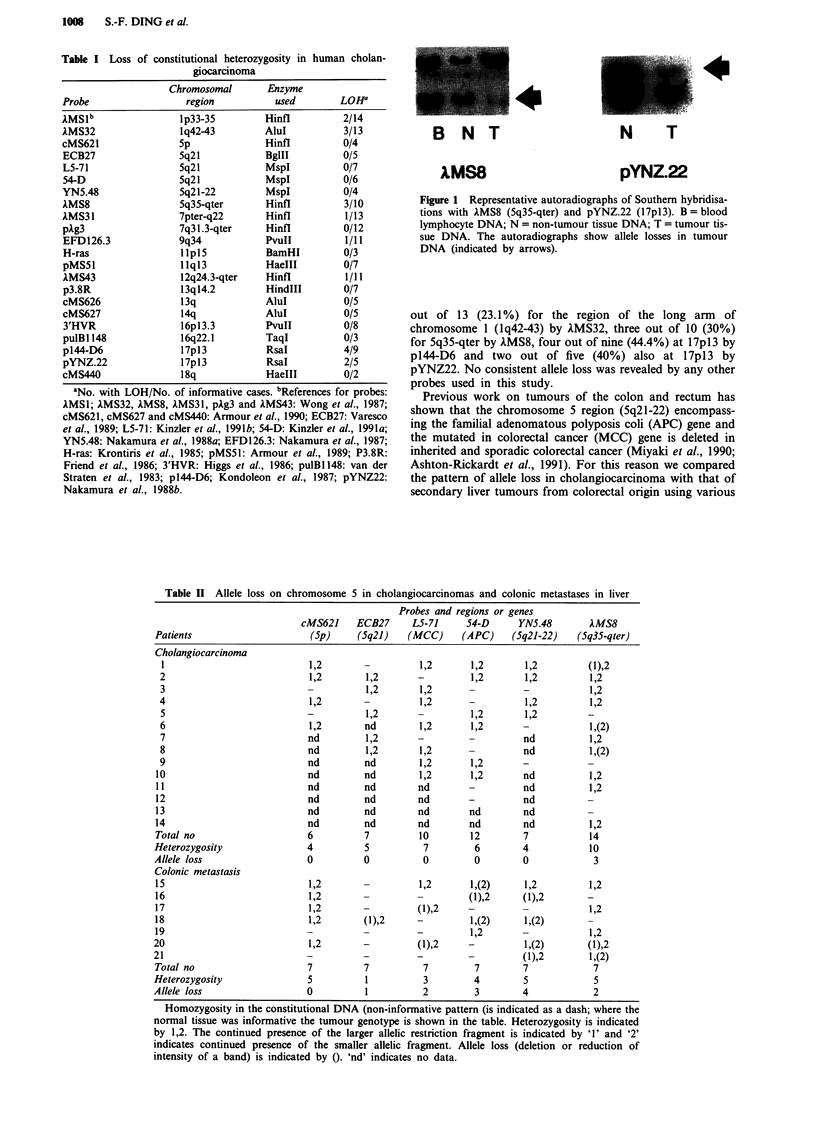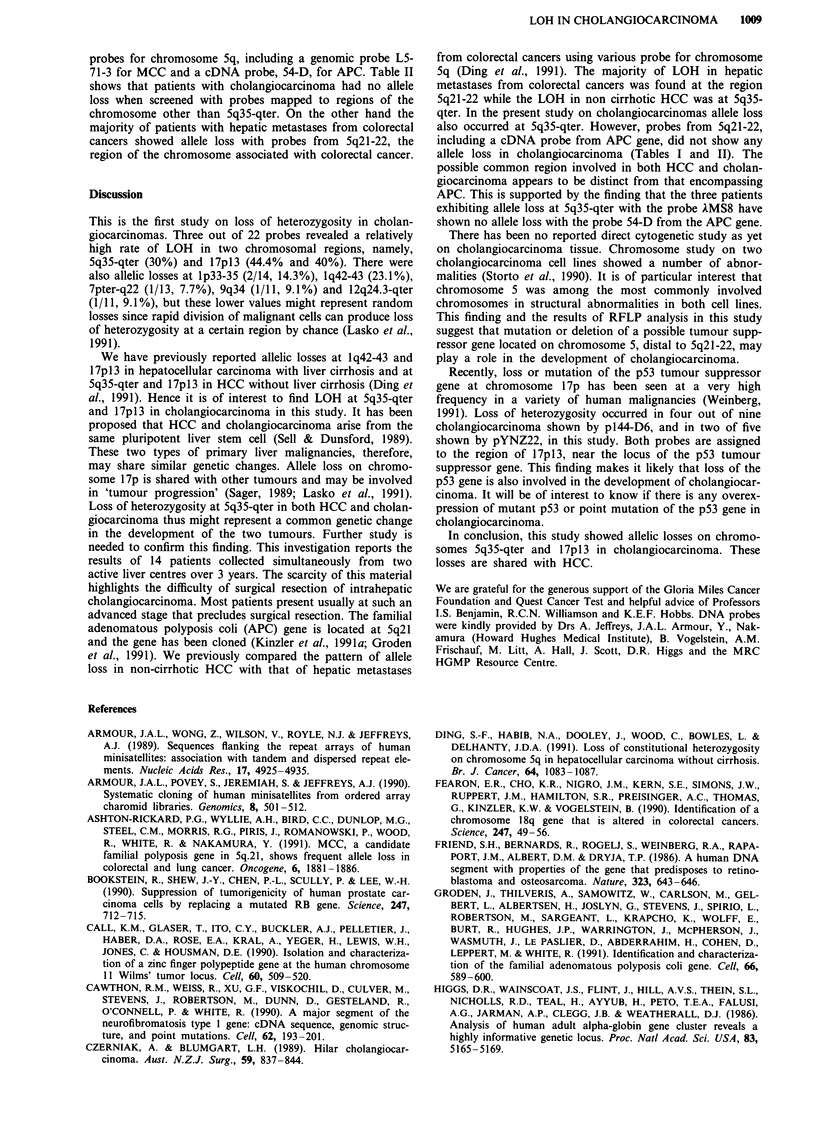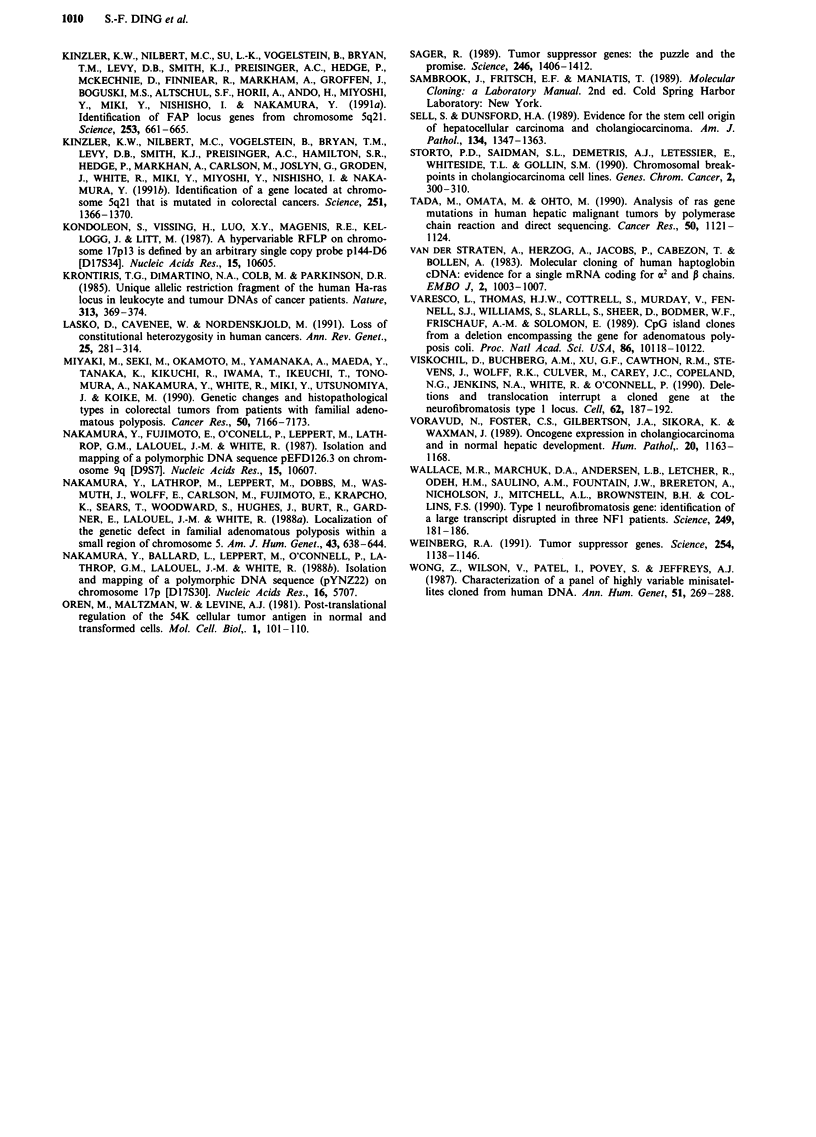# Loss of constitutional heterozygosity on chromosomes 5 and 17 in cholangiocarcinoma.

**DOI:** 10.1038/bjc.1993.184

**Published:** 1993-05

**Authors:** S. F. Ding, J. D. Delhanty, L. Bowles, J. S. Dooley, C. B. Wood, N. A. Habib

**Affiliations:** Department of Surgery, Royal Postgraduate Medical School, Hammersmith Hospital, London, UK.

## Abstract

**Images:**


Br. J. Cancer (1993), 67, 1007-1010 © Macmillan Press Ltd., 1993

### Loss of constitutional heterozygosity on chromosomes 5 and 17 in cholangiocarcinoma

S.-F. Ding[1,2], J.D.A. Delhanty[3], L. Bowles[3], J.S. Dooley[4], C.B. Wood[1] & N.A. Habib[1,2]

[1]*Department of Surgery, Royal Postgraduate Medical School, Hammersmith Hospital, Du Cane Road, London W12 0NN; University Departments of* [2]*Surgery and* [4]*Medicine, Royal Free Hospital School of Medicine, Pond Street, London NW3 2QG;* [3]*Department of Genetics and Biometry, University College London, 4 Stephenson Way, London NW1 2HE, UK.*

**Summary** It has been established that loss of tumour suppressor genes is crucial in carcinogenesis. There has been no reported study on searching for tumour suppressor genes in cholangiocarcinomas as yet. In order to investigate the loss of heterozygosity (LOH), which may represent such gene loss, in cholangiocarcinoma, we studied 14 patients with this tumour using restriction fragment length polymorphism analysis. Twenty-two probes assigned to chromosomes 1, 5, 7, 9, 11, 12, 13, 14, 16, 17 and 18 were used. Allelic losses were found in chromosomal regions 5q35-qter and 17p13. Loss of genetic material in these regions in cholangiocarcinoma was shared with hepatocellular carcinoma. Probes for other chromosomes have as yet shown no consistent LOH. In conclusion, this study for the first time showed LOH on chromosomes 5 and 17 in cholangiocarcinoma.

Cholangiocarcinoma, the intrahepatic bile duct carcinoma, is thought to arise from the same stem cell as hepatocellular carcinoma (HCC) (Sell & Dunsford, 1989). Cholangiocarcinoma is reported as occurring less frequently than HCC in most parts of the world. The prognosis of cholangiocarcinoma is poor, with the majority of patients dying 6-12 months after diagnosis. The overall survival rate in treated cases at 5 years is below 9% (Czerniak & Blumgart, 1989).

There is a growing realisation that cancer is a set of fundamentally genetic diseases (Lasko *et al.*, 1991). Muliple genetic alterations including the activation of oncogenes and the inactivation of tumour suppressor genes are important in carcinogenesis. Tumour suppressor genes are normal cellular genes whose products are thought to be inhibitors of the uncontrolled cellular proliferation characteristic of cancer. Several tumour suppressor genes have been cloned, including the RB1 (Friend *et al.*, 1986), p53 (Oren *et al.*, 1981), WT1 (Call *et al.*, 1990), NF1 (Wallace *et al.*, 1990; Viskochil *et al.*, 1990; Cawthon *et al.*, 1990) and APC (Kinzler *et al.*, 1991*a*; Groden *et al.*, 1991) genes. DCC was clones and could prove to be a candidate suppressor gene (Fearon *et al.*, 1990). Introduction of a normal tumour suppressor gene, for example the RB1 gene, into tumour cells can inhibit tumorigenesis (Bookstein *et al.*, 1990).

Inactivation of tumour suppressor genes can occur via a variety of mechanisms including allele loss and mutation. One of the most widely used techniques for detection of tumour suppressor gene loss is the demonstration of consistent allele loss or loss of heterozygosity (LOH), in tumour cells. This is achieved by using a battery of restriction fragment length polymorphism (RFLP) probes to analyse DNAs from paired samples of non-tumour and tumour tissues (Lasko *et al.*, 1991). A variety of tumours, including both childhood and common adult malignancies, exhibit LOH (Lasko *et al.*, 1991).

Expression of oncogenes, including ras, myc and erbB-2, and point mutations at K-ras codons 12 and 61 have been reported in a high proportion of cholangiocarcinomas (Voravud *et al.*, 1989; Tada *et al.*, 1990). Cytogenetic studies on two cholangiocarcinoma cell lines revealed several chromosomal abnormalities (Storto *et al.*, 1990). To our knowledge, however, there has been no reported study of loss of heterozygosity in cholangiocarcinomas as yet. Here we report the first study of LOH in cholangiocarcinoma with 22 RFLP probes assigned to 11 chromosomes.

### Materials and methods

### *Patients and biopsies*

Fourteen patients with cholangiocarcinoma were studied. All underwent resection of their tumours. None of the patients received chemotherapy or radiotherapy before surgery. Surgical biopsies from tumoral and non-tumoral liver tissues were snap frozen in liquid nitrogen at the time of operation. Lymphocytes from peripheral blood obtained pre-operatively were also used as a source of normal DNA. Tissue was stored at − 70°C until DNA extraction. A portion of each tumour sample was examined histologically to confirm the type of tumour present.

### *DNA extraction and analysis*

DNA was prepared from blood and tissue samples by standard phenol/chloroform methods (Sambrook *et al.*, 1989). Southern analyses were done as previously described (Ding *et al.*, 1991). The 22 RFLP probes for chromosomes 1, 5, 7, 9, 11, 12, 13, 14, 16, 17 and 18 and the appropriate restriction enzymes are listed in Table I. If two alleles appeared as two separate bands in the resultant autoradiograph of the constitutional DNA, the patient was considered 'informative', or heterozygous, for the particular marker. Complete deletion or loss of intensity of one band in the tumour DNA indicated an allele loss, or an LOH. The loss of band intensity was confirmed by examination of the autoradiographs with densitometry. A cutoff level of 50% or more of allele intensity was considered as evidence of LOH.

### Results

Table I shows the overall pattern of allele loss in cholangiocarcinoma. Overall, 164/229 Southern blots were informative (heterozygosity: 71.6%) and the overall LOH was 17 out of 164 informative cases (10.4%). Figure 1 shows representative examples of allele loss.

As shown in Table I, the 14 cholangiocarcinomas had a higher rate of LOH on chromosomes 5 and 17 than on other chromosomes. Allelic losses were shown in two out of 14 informative cases (14.3%) for the region of the short arm of chromosome 1 (1p33-35) detected by the probe λMS1, three

Correspondence: N.A. Habib, Department of Surgery, Royal Postgraduate Medical School, Du Cane Road, London W12 0NN, UK.
Received 18 November 1992.

**Table I** Loss of constitutional heterozygosity in human cholangiocarcinoma

| *Probe* | *Chromosomal region* | *Enzyme used* | *LOH*[a] |
|---|---|---|---|
| λMS1[b] | 1p33-35 | HinfI | 2/14 |
| λMS32 | 1q42-43 | AluI | 3/13 |
| cMS621 | 5p | HinfI | 0/4 |
| ECB27 | 5q21 | BglII | 0/5 |
| L5-71 | 5q21 | MspI | 0/7 |
| 54-D | 5q21 | MspI | 0/6 |
| YN5.48 | 5q21-22 | MspI | 0/4 |
| λMS8 | 5q35-qter | HinfI | 3/10 |
| λMS31 | 7pter-q22 | HinfI | 1/13 |
| pλg3 | 7q31.3-qter | HinfI | 0/12 |
| EFD126.3 | 9q34 | PvuII | 1/11 |
| H-ras | 11p15 | BamHI | 0/3 |
| pMS51 | 11q13 | HaeIII | 0/7 |
| λMS43 | 12q24.3-qter | HinfI | 1/11 |
| p3.8R | 13q14.2 | HindIII | 0/7 |
| cMS626 | 13q | AluI | 0/5 |
| cMS627 | 14q | AluI | 0/5 |
| 3'HVR | 16p13.3 | PvuII | 0/8 |
| pulB1148 | 16q22.1 | TaqI | 0/3 |
| p144-D6 | 17p13 | RsaI | 4/9 |
| pYNZ.22 | 17p13 | RsaI | 2/5 |
| cMS440 | 18q | HaeIII | 0/2 |

[a]No. with LOH/No. of informative cases. [b]References for probes: λMS1; λMS32, λMS8, λMS31, pλg3 and λMS43: Wong *et al.*, 1987; cMS621, cMS627 and cMS440: Armour *et al.*, 1990; ECB27: Varesco *et al.*, 1989; L5-71: Kinzler *et al.*, 1991*b*; 54-D: Kinzler *et al.*, 1991*a*; YN5.48: Nakamura *et al.*, 1988*a*; EFD126.3: Nakamura *et al.*, 1987; H-ras: Krontiris *et al.*, 1985; pMS51: Armour *et al.*, 1989; P3.8R: Friend *et al.*, 1986; 3'HVR: Higgs *et al.*, 1986; pulB1148: van der Straten *et al.*, 1983; p144-D6; Kondoleon *et al.*, 1987; pYNZ22: Nakamura *et al.*, 1988*b*.

**Figure 1** Representative autoradiographs of Southern hybridisations with λMS8 (5q35-qter) and pYNZ.22 (17p13). B = blood lymphocyte DNA; N = non-tumour tissue DNA; T = tumour tissue DNA. The autoradiographs show allele losses in tumour DNA (indicated by arrows).

out of 13 (23.1%) for the region of the long arm of chromosome 1 (1q42-43) by λMS32, three out of 10 (30%) for 5q35-qter by λMS8, four out of nine (44.4%) at 17p13 by p144-D6 and two out of five (40%) also at 17p13 by pYNZ22. No consistent allele loss was revealed by any other probes used in this study.

Previous work on tumours of the colon and rectum has shown that the chromosome 5 region (5q21-22) encompassing the familial adenomatous polyposis coli (APC) gene and the mutated in colorectal cancer (MCC) gene is deleted in inherited and sporadic colorectal cancer (Miyaki *et al.*, 1990; Ashton-Rickardt *et al.*, 1991). For this reason we compared the pattern of allele loss in cholangiocarcinoma with that of secondary liver tumours from colorectal origin using various

**Table II** Allele loss on chromosome 5 in cholangiocarcinomas and colonic metastases in liver

| | *Probes and regions or genes* | | | | | |
|---|---|---|---|---|---|---|
| *Patients* | *cMS621 (5p)* | *ECB27 (5q21)* | *L5-71 (MCC)* | *54-D (APC)* | *YN5.48 (5q21-22)* | *λMS8 (5q35-qter)* |
| *Cholangiocarcinoma* | | | | | | |
| 1 | 1,2 | – | 1,2 | 1,2 | 1,2 | (1),2 |
| 2 | 1,2 | 1,2 | – | 1,2 | 1,2 | 1,2 |
| 3 | – | 1,2 | 1,2 | – | – | 1,2 |
| 4 | 1,2 | – | 1,2 | – | 1,2 | 1,2 |
| 5 | – | 1,2 | – | 1,2 | 1,2 | – |
| 6 | 1,2 | nd | 1,2 | 1,2 | – | 1,(2) |
| 7 | nd | 1,2 | – | – | nd | 1,2 |
| 8 | nd | 1,2 | 1,2 | – | nd | 1,(2) |
| 9 | nd | nd | 1,2 | 1,2 | – | – |
| 10 | nd | nd | 1,2 | 1,2 | nd | 1,2 |
| 11 | nd | nd | nd | – | nd | 1,2 |
| 12 | nd | nd | nd | – | nd | – |
| 13 | nd | nd | nd | nd | nd | – |
| 14 | nd | nd | nd | nd | nd | 1,2 |
| *Total no* | 6 | 7 | 10 | 12 | 7 | 14 |
| *Heterozygosity* | 4 | 5 | 7 | 6 | 4 | 10 |
| *Allele loss* | 0 | 0 | 0 | 0 | 0 | 3 |
| *Colonic metastasis* | | | | | | |
| 15 | 1,2 | – | 1,2 | 1,(2) | 1,2 | 1,2 |
| 16 | 1,2 | – | – | (1),2 | (1),2 | – |
| 17 | 1,2 | – | (1),2 | – | – | 1,2 |
| 18 | 1,2 | (1),2 | – | 1,(2) | 1,(2) | – |
| 19 | – | – | – | 1,2 | – | 1,2 |
| 20 | 1,2 | – | (1),2 | – | 1,(2) | (1),2 |
| 21 | – | – | – | – | (1),2 | 1,(2) |
| *Total no* | 7 | 7 | 7 | 7 | 7 | 7 |
| *Heterozygosity* | 5 | 1 | 3 | 4 | 5 | 5 |
| *Allele loss* | 0 | 1 | 2 | 3 | 4 | 2 |

Homozygosity in the constitutional DNA (non-informative pattern (is indicated as a dash; where the normal tissue was informative the tumour genotype is shown in the table. Heterozygosity is indicated by 1,2. The continued presence of the larger allelic restriction fragment is indicated by '1' and '2' indicates continued presence of the smaller allelic fragment. Allele loss (deletion or reduction of intensity of a band) is indicated by (). 'nd' indicates no data.

probes for chromosome 5q, including a genomic probe L5-71-3 for MCC and a cDNA probe, 54-D, for APC. Table II shows that patients with cholangiocarcinoma had no allele loss when screened with probes mapped to regions of the chromosome other than 5q35-qter. On the other hand the majority of patients with hepatic metastases from colorectal cancers showed allele loss with probes from 5q21-22, the region of the chromosome associated with colorectal cancer.

### Discussion

This is the first study on loss of heterozygosity in cholangiocarcinomas. Three out of 22 probes revealed a relatively high rate of LOH in two chromosomal regions, namely, 5q35-qter (30%) and 17p13 (44.4% and 40%). There were also allelic losses at 1p33-35 (2/14, 14.3%), 1q42-43 (23.1%), 7pter-q22 (1/13, 7.7%), 9q34 (1/11, 9.1%) and 12q24.3-qter (1/11, 9.1%), but these lower values might represent random losses since rapid division of malignant cells can produce loss of heterozygosity at a certain region by chance (Lasko *et al.*, 1991).

We have previously reported allelic losses at 1q42-43 and 17p13 in hepatocellular carcinoma with liver cirrhosis and at 5q35-qter and 17p13 in HCC without liver cirrhosis (Ding *et al.*, 1991). Hence it is of interest to find LOH at 5q35-qter and 17p13 in cholangiocarcinoma in this study. It has been proposed that HCC and cholangiocarcinoma arise from the same pluripotent liver stem cell (Sell & Dunsford, 1989). These two types of primary liver malignancies, therefore, may share similar genetic changes. Allele loss on chromosome 17p is shared with other tumours and may be involved in 'tumour progression' (Sager, 1989; Lasko *et al.*, 1991). Loss of heterozygosity at 5q35-qter in both HCC and cholangiocarcinoma thus might represent a common genetic change in the development of the two tumours. Further study is needed to confirm this finding. This investigation reports the results of 14 patients collected simultaneously from two active liver centres over 3 years. The scarcity of this material highlights the difficulty of surgical resection of intrahepatic cholangiocarcinoma. Most patients present usually at such an advanced stage that precludes surgical resection. The familial adenomatous polyposis coli (APC) gene is located at 5q21 and the gene has been cloned (Kinzler *et al.*, 1991*a*; Groden *et al.*, 1991). We previously compared the pattern of allele loss in non-cirrhotic HCC with that of hepatic metastases from colorectal cancers using various probe for chromosome 5q (Ding *et al.*, 1991). The majority of LOH in hepatic metastases from colorectal cancers was found at the region 5q21-22 while the LOH in non cirrhotic HCC was at 5q35-qter. In the present study on cholangiocarcinomas allele loss also occurred at 5q35-qter. However, probes from 5q21-22, including a cDNA probe from APC gene, did not show any allele loss in cholangiocarcinoma (Tables I and II). The possible common region involved in both HCC and cholangiocarcinoma appears to be distinct from that encompassing APC. This is supported by the finding that the three patients exhibiting allele loss at 5q35-qter with the probe λMS8 have shown no allele loss with the probe 54-D from the APC gene.

There has been no reported direct cytogenetic study as yet on cholangiocarcinoma tissue. Chromosome study on two cholangiocarcinoma cell lines showed a number of abnormalities (Storto *et al.*, 1990). It is of particular interest that chromosome 5 was among the most commonly involved chromosomes in structural abnormalities in both cell lines. This finding and the results of RFLP analysis in this study suggest that mutation or deletion of a possible tumour suppressor gene located on chromosome 5, distal to 5q21-22, may play a role in the development of cholangiocarcinoma.

Recently, loss or mutation of the p53 tumour suppressor gene at chromosome 17p has been seen at a very high frequency in a variety of human malignancies (Weinberg, 1991). Loss of heterozygosity occurred in four out of nine cholangiocarcinoma shown by p144-D6, and in two of five shown by pYNZ22, in this study. Both probes are assigned to the region of 17p13, near the locus of the p53 tumour suppressor gene. This finding makes it likely that loss of the p53 gene is also involved in the development of cholangiocarcinoma. It will be of interest to know if there is any overexpression of mutant p53 or point mutation of the p53 gene in cholangiocarcinoma.

In conclusion, this study showed allelic losses on chromosomes 5q35-qter and 17p13 in cholangiocarcinoma. These losses are shared with HCC.

We are grateful for the generous support of the Gloria Miles Cancer Foundation and Quest Cancer Test and helpful advice of Professors I.S. Benjamin, R.C.N. Williamson and K.E.F. Hobbs. DNA probes were kindly provided by Drs A. Jeffreys, J.A.L. Armour, Y., Nakamura (Howard Hughes Medical Institute), B. Vogelstein, A.M. Frischauf, M. Litt, A. Hall, J. Scott, D.R. Higgs and the MRC HGMP Resource Centre.

### References

ARMOUR, J.A.L., WONG, Z., WILSON, V., ROYLE, N.J. & JEFFREYS, A.J. (1989). Sequences flanking the repeat arrays of human minisatellites: association with tandem and dispersed repeat elements. *Nucleic Acids Res.*, **17**, 4925-4935.

ARMOUR, J.A.L., POVEY, S., JEREMIAH, S. & JEFFREYS, A.J. (1990). Systematic cloning of human minisatellites from ordered array charomid libraries. *Genomics*, **8**, 501-512.

ASHTON-RICKARD, P.G., WYLLIE, A.H., BIRD, C.C., DUNLOP, M.G., STEEL, C.M., MORRIS, R.G., PIRIS, J., ROMANOWSKI, P., WOOD, R., WHITE, R. & NAKAMURA, Y. (1991). MCC, a candidate familial polyposis gene in 5q.21, shows frequent allele loss in colorectal and lung cancer. *Oncogene*, **6**, 1881-1886.

BOOKSTEIN, R., SHEW, J.-Y., CHEN, P.-L., SCULLY, P. & LEE, W.-H. (1990). Suppression of tumorigenicity of human prostate carcinoma cells by replacing a mutated RB gene. *Science*, **247**, 712-715.

CALL, K.M., GLASER, T., ITO, C.Y., BUCKLER, A.J., PELLETIER, J., HABER, D.A., ROSE, E.A., KRAL, A., YEGER, H., LEWIS, W.H., JONES, C. & HOUSMAN, D.E. (1990). Isolation and characterization of a zinc finger polypeptide gene at the human chromosome 11 Wilms' tumor locus. *Cell*, **60**, 509-520.

CAWTHON, R.M., WEISS, R., XU, G.F., VISKOCHIL, D., CULVER, M., STEVENS, J., ROBERTSON, M., DUNN, D., GESTELAND, R., O'CONNELL, P. & WHITE, R. (1990). A major segment of the neurofibromatosis type 1 gene: cDNA sequence, genomic structure, and point mutations. *Cell*, **62**, 193-201.

CZERNIAK, A. & BLUMGART, L.H. (1989). Hilar cholangiocarcinoma. *Aust. N.Z.J. Surg.*, **59**, 837-844.

DING, S.-F., HABIB, N.A., DOOLEY, J., WOOD, C., BOWLES, L. & DELHANTY, J.D.A. (1991). Loss of constitutional heterozygosity on chromosome 5q in hepatocellular carcinoma without cirrhosis. *Br. J. Cancer*, **64**, 1083-1087.

FEARON, E.R., CHO, K.R., NIGRO, J.M., KERN, S.E., SIMONS, J.W., RUPPERT, J.M., HAMILTON, S.R., PREISINGER, A.C., THOMAS, G., KINZLER, K.W. & VOGELSTEIN, B. (1990). Identification of a chromosome 18q gene that is altered in colorectal cancers. *Science*, **247**, 49-56.

FRIEND, S.H., BERNARDS, R., ROGELJ, S., WEINBERG, R.A., RAPAPORT, J.M., ALBERT, D.M. & DRYJA, T.P. (1986). A human DNA segment with properties of the gene that predisposes to retinoblastoma and osteosarcoma. *Nature*, **323**, 643-646.

GRODEN, J., THILVERIS, A., SAMOWITZ, W., CARLSON, M., GELBERT, L., ALBERTSEN, H., JOSLYN, G., STEVENS, J., SPIRIO, L., ROBERTSON, M., SARGEANT, L., KRAPCHO, K., WOLFF, E., BURT, R., HUGHES, J.P., WARRINGTON, J., MCPHERSON, J., WASMUTH, J., LE PASLIER, D., ABDERRAHIM, H., COHEN, D., LEPPERT, M. & WHITE, R. (1991). Identification and characterization of the familial adenomatous polyposis coli gene. *Cell*, **66**, 589-600.

HIGGS, D.R., WAINSCOAT, J.S., FLINT, J., HILL, A.V.S., THEIN, S.L., NICHOLLS, R.D., TEAL, H., AYYUB, H., PETO, T.E.A., FALUSI, A.G., JARMAN, A.P., CLEGG, J.B. & WEATHERALL, D.J. (1986). Analysis of human adult alpha-globin gene cluster reveals a highly informative genetic locus. *Proc. Natl Acad. Sci. USA*, **83**, 5165-5169.

KINZLER, K.W., NILBERT, M.C., SU, L.-K., VOGELSTEIN, B., BRYAN, T.M., LEVY, D.B., SMITH, K.J., PREISINGER, A.C., HEDGE, P., MCKECHNIE, D., FINNIEAR, R., MARKHAM, A., GROFFEN, J., BOGUSKI, M.S., ALTSCHUL, S.F., HORII, A., ANDO, H., MIYOSHI, Y., MIKI, Y., NISHISHO, I. & NAKAMURA, Y. (1991*a*). Identification of FAP locus genes from chromosome 5q21. *Science*, **253**, 661–665.

KINZLER, K.W., NILBERT, M.C., VOGELSTEIN, B., BRYAN, T.M., LEVY, D.B., SMITH, K.J., PREISINGER, A.C., HAMILTON, S.R., HEDGE, P., MARKHAN, A., CARLSON, M., JOSLYN, G., GRODEN, J., WHITE, R., MIKI, Y., MIYOSHI, Y., NISHISHO, I. & NAKAMURA, Y. (1991*b*). Identification of a gene located at chromosome 5q21 that is mutated in colorectal cancers. *Science*, **251**, 1366–1370.

KONDOLEON, S., VISSING, H., LUO, X.Y., MAGENIS, R.E., KELLOGG, J. & LITT, M. (1987). A hypervariable RFLP on chromosome 17p13 is defined by an arbitrary single copy probe p144-D6 [D17S34]. *Nucleic Acids Res.*, **15**, 10605.

KRONTIRIS, T.G., DIMARTINO, N.A., COLB, M. & PARKINSON, D.R. (1985). Unique allelic restriction fragment of the human Ha-ras locus in leukocyte and tumour DNAs of cancer patients. *Nature*, **313**, 369–374.

LASKO, D., CAVENEE, W. & NORDENSKJOLD, M. (1991). Loss of constitutional heterozygosity in human cancers. *Ann. Rev. Genet.*, **25**, 281–314.

MIYAKI, M., SEKI, M., OKAMOTO, M., YAMANAKA, A., MAEDA, Y., TANAKA, K., KIKUCHI, R., IWAMA, T., IKEUCHI, T., TONOMURA, A., NAKAMURA, Y., WHITE, R., MIKI, Y., UTSUNOMIYA, J. & KOIKE, M. (1990). Genetic changes and histopathological types in colorectal tumors from patients with familial adenomatous polyposis. *Cancer Res.*, **50**, 7166–7173.

NAKAMURA, Y., FUJIMOTO, E., O'CONELL, P., LEPPERT, M., LATHROP, G.M., LALOUEL, J.-M. & WHITE, R. (1987). Isolation and mapping of a polymorphic DNA sequence pEFD126.3 on chromosome 9q [D9S7]. *Nucleic Acids Res.*, **15**, 10607.

NAKAMURA, Y., LATHROP, M., LEPPERT, M., DOBBS, M., WASMUTH, J., WOLFF, E., CARLSON, M., FUJIMOTO, E., KRAPCHO, K., SEARS, T., WOODWARD, S., HUGHES, J., BURT, R., GARDNER, E., LALOUEL, J.-M. & WHITE, R. (1988*a*). Localization of the genetic defect in familial adenomatous polyposis within a small region of chromosome 5. *Am. J. Hum. Genet.*, **43**, 638–644.

NAKAMURA, Y., BALLARD, L., LEPPERT, M., O'CONNELL, P., LATHROP, G.M., LALOUEL, J.-M. & WHITE, R. (1988*b*). Isolation and mapping of a polymorphic DNA sequence (pYNZ22) on chromosome 17p [D17S30]. *Nucleic Acids Res.*, **16**, 5707.

OREN, M., MALTZMAN, W. & LEVINE, A.J. (1981). Post-translational regulation of the 54K cellular tumor antigen in normal and transformed cells. *Mol. Cell. Biol,*. **1**, 101–110.

SAGER, R. (1989). Tumor suppressor genes: the puzzle and the promise. *Science*, **246**, 1406–1412.

SAMBROOK, J., FRITSCH, E.F. & MANIATIS, T. (1989). *Molecular Cloning: a Laboratory Manual.* 2nd ed. Cold Spring Harbor Laboratory: New York.

SELL, S. & DUNSFORD, H.A. (1989). Evidence for the stem cell origin of hepatocellular carcinoma and cholangiocarcinoma. *Am. J. Pathol.*, **134**, 1347–1363.

STORTO, P.D., SAIDMAN, S.L., DEMETRIS, A.J., LETESSIER, E., WHITESIDE, T.L. & GOLLIN, S.M. (1990). Chromosomal breakpoints in cholangiocarcinoma cell lines. *Genes. Chrom. Cancer*, **2**, 300–310.

TADA, M., OMATA, M. & OHTO, M. (1990). Analysis of ras gene mutations in human hepatic malignant tumors by polymerase chain reaction and direct sequencing. *Cancer Res.*, **50**, 1121–1124.

VAN DER STRATEN, A., HERZOG, A., JACOBS, P., CABEZON, T. & BOLLEN, A. (1983). Molecular cloning of human haptoglobin cDNA: evidence for a single mRNA coding for $\alpha^2$ and $\beta$ chains. *EMBO J*, **2**, 1003–1007.

VARESCO, L., THOMAS, H.J.W., COTTRELL, S., MURDAY, V., FENNELL, S.J., WILLIAMS, S., SLARLL, S., SHEER, D., BODMER, W.F., FRISCHAUF, A.-M. & SOLOMON, E. (1989). CpG island clones from a deletion encompassing the gene for adenomatous polyposis coli. *Proc. Natl Acad. Sci. USA*, **86**, 10118–10122.

VISKOCHIL, D., BUCHBERG, A.M., XU, G.F., CAWTHON, R.M., STEVENS, J., WOLFF, R.K., CULVER, M., CAREY, J.C., COPELAND, N.G., JENKINS, N.A., WHITE, R. & O'CONNELL, P. (1990). Deletions and translocation interrupt a cloned gene at the neurofibromatosis type 1 locus. *Cell*, **62**, 187–192.

VORAVUD, N., FOSTER, C.S., GILBERTSON, J.A., SIKORA, K. & WAXMAN, J. (1989). Oncogene expression in cholangiocarcinoma and in normal hepatic development. *Hum. Pathol,*. **20**, 1163–1168.

WALLACE, M.R., MARCHUK, D.A., ANDERSEN, L.B., LETCHER, R., ODEH, H.M., SAULINO, A.M., FOUNTAIN, J.W., BRERETON, A., NICHOLSON, J., MITCHELL, A.L., BROWNSTEIN, B.H. & COLLINS, F.S. (1990). Type 1 neurofibromatosis gene: identification of a large transcript disrupted in three NF1 patients. *Science*, **249**, 181–186.

WEINBERG, R.A. (1991). Tumor suppressor genes. *Science*, **254**, 1138–1146.

WONG, Z., WILSON, V., PATEL, I., POVEY, S. & JEFFREYS, A.J. (1987). Characterization of a panel of highly variable minisatellites cloned from human DNA. *Ann. Hum. Genet*, **51**, 269–288.